# Social media use, smoking expectancies, and nicotine experimentation in early adolescents: A prospective cohort study

**DOI:** 10.1111/ajad.70135

**Published:** 2026-02-10

**Authors:** Jason M. Nagata, Andrew Caffrey, Alexander Heuer, Keira Beltran Murillo, Christiane K. Helmer, Isaac Frimpong, Colbey Ricklefs, Abubakr A. Al‐Shoaibi, Alexander Testa, Claire D. Brindis, Glenn‐Milo Santos, Fiona C. Baker

**Affiliations:** ^1^ Department of Pediatrics University of California, San Francisco San Francisco California USA; ^2^ Philip R. Lee Institute for Health Policy Studies University of California, San Francisco San Francisco California USA; ^3^ Department of Management, Policy and Community Health University of Texas Health Science Center at Houston Houston Texas USA; ^4^ Department of Community Health Systems, School of Nursing University of California, San Francisco San Francisco California USA; ^5^ Center for Health Sciences SRI International Menlo Park California USA

## Abstract

**Background and Objectives:**

Social media exposure may influence early nicotine experimentation, a behavior linked to later nicotine dependence and health risks. Few studies have examined the role of smoking expectancies (i.e., beliefs about the anticipated positive or negative effects of nicotine) as a pathway underlying this association, especially in early adolescence. The objective of this study is to examine the prospective association between social media use and nicotine experimentation in early adolescence, and whether smoking expectancies mediate this relationship.

**Methods:**

Using longitudinal data from the Adolescent Brain Cognitive Development Study (*N* = 8292; mean age 12.0 years at Year 2; 2018–2020), we estimated associations between social media time (Year 2) and nicotine experimentation (Year 4), adjusting for confounders and testing positive and negative smoking expectancies (Year 3) as mediators using generalized structural equation modeling.

**Results:**

Social media time at Year 2 was associated with nicotine experimentation at Year 4. Positive smoking expectancies (but not negative expectancies) were associated with nicotine experimentation. Positive smoking expectancies mediated 5.97% (95% CI: 1.27%–10.67%, *p* = .013) of the social media‐nicotine experimentation association.

**Discussion and Conclusions:**

Early social media exposure may be associated with favorable beliefs about nicotine, increasing adolescents' risk of experimentation. Regulatory policies, clinical screening, and prevention programs could mitigate early nicotine use. Future research should explore how these relationships evolve across adolescence.

**Scientific Significance:**

This study advances understanding of how social media use contributes to early nicotine experimentation in adolescents by identifying positive smoking expectancies as a potential pathway.

## INTRODUCTION

Nicotine use remains a major public health concern for adolescents. Although cigarette smoking has declined, the use of electronic nicotine delivery systems (e‐cigarettes) has increased, with more adolescents in the United States (U.S.) now reporting e‐cigarette use than traditional cigarette use.^1^ For example, among middle and high school students, e‐cigarettes were the most commonly reported tobacco or nicotine product in 2024, with 5.9% reporting use in the past 30 days, compared to 1.4% for traditional cigarettes.^2^ Early e‐cigarette exposure is associated with later cigarette smoking and sustained nicotine dependence.^3^ Greater e‐cigarette use among young people has raised concerns about related health risks, including exposure to carcinogens like formaldehyde and flavorings such as diacetyl, which have been linked to lung disease.^4^

Expectancies, or beliefs about the effects of nicotine use, play a central role in shaping behavior.^5^ Positive expectancies, such as beliefs that nicotine use reduces stress or enhances social experiences, are linked to a higher likelihood of use, while negative expectancies, such as beliefs that nicotine impairs health or warrants social disproval, may discourage use.^5^ These expectancies may be shaped by social media, as observing others' behaviors in social contexts can shape adolescents' perceptions of nicotine use according to Social Learning Theory.^6^ Around 69.5% of adolescents use social media platforms, including 63.8% of children under age 13,^7^ which frequently feature nicotine‐related content.^5^, ^8^ For example, 24% of young adults reported seeing e‐cigarette posts on Facebook and 20% reported seeing them on Instagram “sometimes” or “often.”^5^, ^8^ Platforms, including Twitter, Instagram, YouTube, and TikTok, display over 76% of substance‐related content in a normalized or positive light.^9^ Glamorized portrayals of nicotine use from peers, influencers, and e‐cigarette companies may reinforce positive expectancies by modeling socially accepted and appealing behaviors.

Several studies have demonstrated that social media exposure to nicotine‐related content is linked to nicotine use outcomes among adolescents.^3^, ^10^, ^11^ Passive exposure to nicotine‐related content on social media can increase the likelihood of nicotine use, with its influence extending beyond portrayals in television and movies and further amplifying the risk of smoking initiation.^8^, ^12^ However, few studies have focused on early adolescence, a critical developmental window characterized by heightened vulnerability to external influences.^13^ According to the Life Course Theory, exposures during this stage can shape long‐term behavioral trajectories, including susceptibility to substance use.^14^ Specifically, both overall social media use and exposure to positive nicotine‐related content on social media can increase the likelihood that adolescents endorse positive expectancies about nicotine use, which in turn, are associated with later nicotine use.^5^, ^10^, ^11^, ^15^

Despite evidence linking social media exposure to nicotine initiation, the majority of studies in this area are cross‐sectional and do not employ mediation analyses. As a result, the role of mechanisms, such as smoking attitudes, in adolescent nicotine experimentation over time remains largely unexplored. To address this gap, the present study examines the pathway from social media exposure to nicotine experimentation, with smoking expectancies as a mediator. The goal is to clarify mechanisms that may inform prevention strategies. We hypothesized that social media time would be associated with a higher risk of nicotine experimentation and that this relationship would be mediated by nicotine expectancies: greater social media use would be associated with stronger positive expectancies and subsequently higher risk for nicotine experimentation, whereas lower social media use would be associated with negative expectancies, reducing the risk for nicotine experimentation.

## METHODS

We used longitudinal data (Years 2–4) from the Adolescent Brain Cognitive Development (ABCD) Study, which recruited a large, diverse sample of 9–10‐year‐olds in the U.S. at baseline (2016–2018) from 21 sites nationwide. Recruitment, procedures, and measures have been previously described.^16^ Participants with missing data for social media use, nicotine variables, or sociodemographic factors were excluded, leaving a total of 8292 participants in the analysis (Appendix A). The University of California, San Diego, provided centralized IRB approval (160091), with local IRB approvals obtained at each site. Caregivers provided written informed consent, and each child provided written assent.

### Independent variable: Social media time

Adolescents reported their usual daily time spent on social media, in hours and minutes, separately for weekdays and weekends, at the Year 2 follow‐up (2018–2020). To calculate overall daily social media use, a weighted average was used: ([weekday average×5] + [weekend average×2])/7.^17^ In the ABCD Study, self‐reported screen time showed a significant, moderate correlation with data from a passive smartphone monitoring app (*r* = 0.49, *p* < .001).^18^

### Dependent variable: Nicotine experimentation

ABCD Study participants used a modified version of the Timeline Follow‐Back (TLFB), a calendar‐based tool for recalling substance use, including nicotine.^19^, ^20^ The TLFB has strong psychometric properties, demonstrating high test‐retest reliability and discriminant and convergent validity,^21^, ^22^ and has been validated for use with adolescents as young as 12 years old.^23^, ^24^ The TLFB has also been shown to have good validity compared with biomarkers such as salivary cotinine in the assessment of adolescent smoking.^25^ Consistent with ABCD protocols for sensitive youth‐reported assessments, substance use measures, including the TLFB interview, were self‐administered by adolescents using an Audio Computer‐Assisted Self‐Interview system to ensure privacy and promote accurate reporting. This was conducted without the presence of a parent or guardian. The current analysis used the TLFB question asking whether children had ever tried a puff of a nicotine product, such as a cigarette, e‐cigarette, or smokeless tobacco, since the last visit (including both first‐time experimenters and those who had experimented previously), which was assessed at Year 4 follow‐up (2020–2022).^19^ Therefore, the measure includes individuals who might have also reported use in earlier visits and people who have used for the first time. The response to this question was dichotomized (yes/no) for our outcome variable, defined as past‐year nicotine experimentation at Year 4.

### Mediator variables: Smoking expectancies

Smoking expectancies were measured using the Adolescent Smoking Consequences Questionnaire, validated for adolescents,^26^, ^27^ modified by asking about 6 items. These 5‐point Likert measures assess positive (e.g., stress reduction, social facilitation, weight control) and negative (e.g., health risks, negative social impression, physical discomfort) expectancies across seven domains. The measures have demonstrated acceptable internal consistency (Cronbach's α = .56–.88) and test–retest reliability in adolescent samples.^27^ Composite sum scores for positive and negative expectancies were calculated for Year 3 (2019–2021) by summing participants' Likert scale responses for each expectancy type (Appendix B).

### Covariates

Based on prior literature,^28^ we adjusted for age, sex (female, male), race/ethnicity (Asian, Black, Latino/Hispanic, Native American, White, other), household income (six categories), highest parent education (high school or less vs. college or more), depressive symptoms via the Child Behavior Checklist, and study site to address potential sociodemographic and mental health confounders. We also adjusted for smoking expectancies and nicotine experimentation at Year 2 to account for prior beliefs and experimentation that could confound the association between the predictor, mediator, and outcome.

### Statistical analysis

Statistical analyses were conducted using Stata 18 (StataCorp). Generalized structural equation modeling (GSEM) was used to assess the mediating effect of nicotine smoking expectancies on the relationship between time spent on social media and nicotine experimentation. GSEM integrates aspects of path analysis, multiple regression, and confirmatory factor analysis to capture how sociodemographic and mediator data influence the outcome. GSEM then quantifies how the multifaceted variables influence the complex direct and indirect pathways leading to the study outcomes by considering the direct, indirect, and total effects of the variables used within this study.^29^ In our application, the outcome variable (past‐year nicotine experimentation at Year 4) was binary and therefore modeled as dichotomous using a binomial distribution with a logit link function. Specifically, the model examined the direct effect of social media time at Year 2 on past‐year nicotine experimentation at Year 4, as well as the indirect effect mediated by smoking expectancies at Year 3, which were modeled in separate pathways. Models estimated the proportion of the total association between social media time and nicotine experimentation that was mediated by positive and negative smoking expectancies, separately. Indirect effects and their standard errors were computed using the delta method. The proportion mediated was calculated as the ratio of the indirect effect to the total effect (direct + indirect). Covariates and sampling weights for the analysis were integrated and applied, matching key sociodemographic variables in the ABCD Study to the American Community Survey.^30^ Two‐sided alpha was set at *p* < .05.

## RESULTS

Of the 8292 participants who met our inclusion criteria, 48.7% were female, 43.3% were racial/ethnic minorities, and the mean age was 12.0 years (SD = 0.7) at Year 2. At Year 4, 5.4% reported having experimented with nicotine in the prior year (Table 1).

**TABLE 1:** Sociodemographic and nicotine characteristics of 8292 Adolescent Brain Cognitive Development (ABCD) Study participants.

Sociodemographic and nicotine characteristics	Mean (SD)/(%)
Age (Year 2, years)	12.0 (0.7)
Sex (baseline)	
Female	48.7%
Male	51.3%
Race and ethnicity (baseline)	
Asian	5.3%
Black	14.4%
Latino/Hispanic	19.0%
Native American	3.2%
Other	1.3%
White	56.7%
Household income (Year 2)	
$24,999 or less	13.5%
$25,000 to $49,999	16.3%
$50,000 to $74,999	16.7%
$75,000 to $99,999	15.1%
$100,000 to $199,999	28.2%
$200,000 or greater	10.3%
Parent's highest education (Year 2)	
High school education or less	10.5%
College education or more	89.5%
CBCL depressive symptoms t‐score (Year 2)	54.4 (6.2)
Social media, hours per day (Year 2)	0.7 (1.5)
Have had a puff of nicotine since past visit	
Yes (Year 2)	0.9%
Yes (Year 4)	5.4%
Smoking expectancy sum score (Year 3)^a^	
Positive	5.6 (2.2)
Negative	7.2 (2.1)

*Note*: Sampling weights were applied to yield representative estimates based on the American Community Survey from the US Census.

Abbreviations: CBCL, Child Behavior Checklist; SD, standard deviation.

^a^ Possible and observed scoring ranges for smoking expectancy sum score: positive expectancies range from 4 to 20; negative expectancies range from 2 to 10.

**FIGURE 1:**
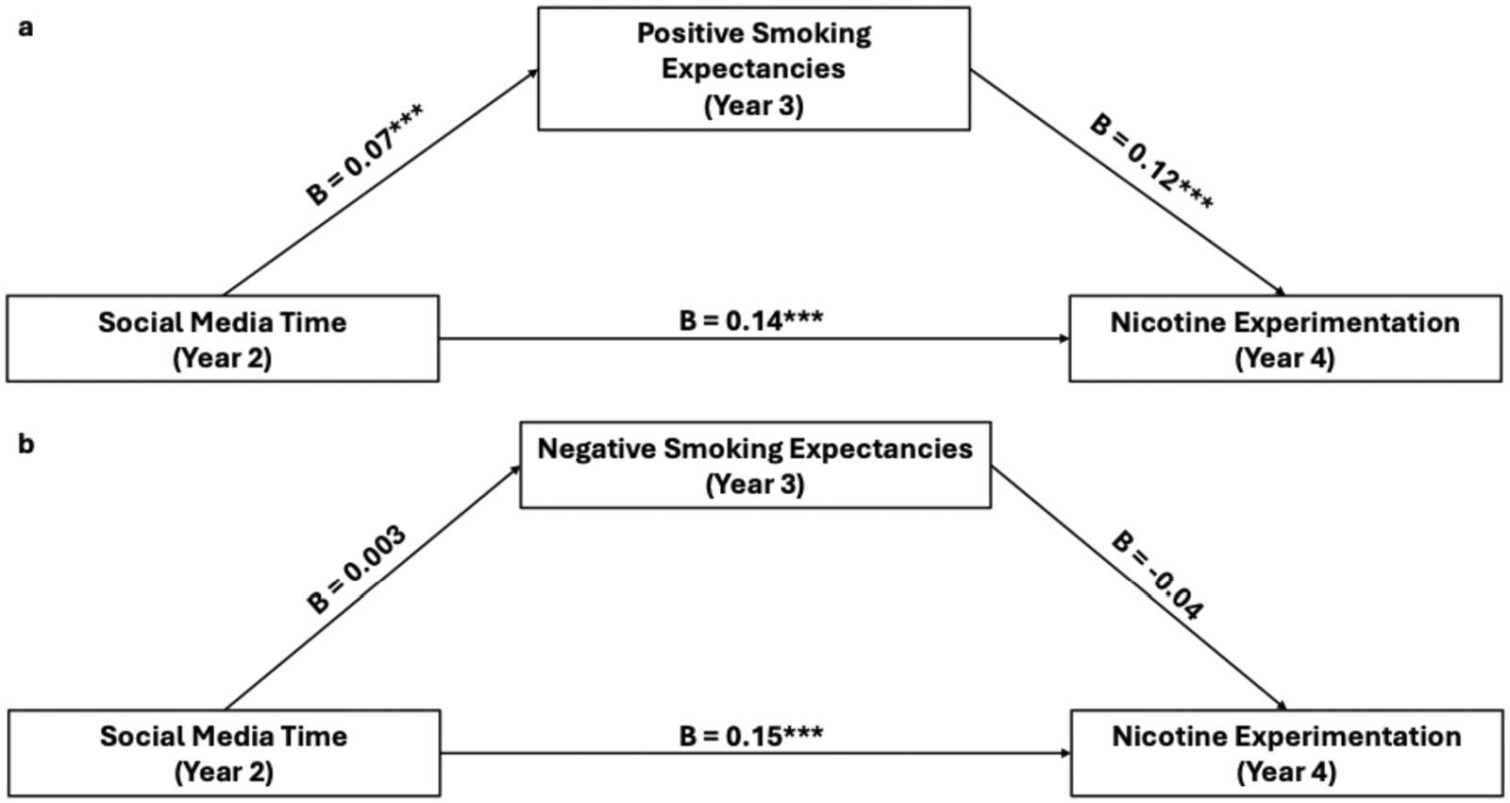
Direct and indirect (via smoking expectancies) effects of social media on nicotine experimentation. Asterisks denote statistical significance: (***) for *p* < .001. (a) Mediation of positive smoking expectancies. (b) Mediation of negative smoking expectancies.

In the model adjusted for positive expectancies, social media use at Year 2 was associated with nicotine experimentation at Year 4 (coefficient [*B*] = 0.14, 95% confidence interval [CI]: 0.08–0.20, *p* < .001) (Figure 1, Table 2). In the model adjusted for negative expectancies, social media use at Year 2 was associated with nicotine experimentation at Year 4 (*B* = 0.15, 95% CI: 0.10–0.21, *p* < .001) (Figure 1, Table 2). Positive smoking expectancies were associated with nicotine experimentation (*B* = 0.12, 95% CI: 0.07–0.17, *p* < .001), while negative smoking expectancies were not associated. Positive smoking expectancies mediated 5.97% (95% CI: 1.27%–10.67%, *p* = .013) of the social media‐nicotine experimentation association, while negative smoking expectancies did not significantly mediate the relationship (Table 2).

**TABLE 2:** Direct and indirect effects of associations of social media time (Year 2) and nicotine experimentation (Year 4) through positive and negative smoking expectancies (Year 3).

		Direct effect			Indirect effect through smoking expectancies			Proportion mediated	
		*B* (SE)	95% CI	*p*	*B* (SE)	95% CI	*p*	*B* (95% CI) (%)	*p*
Social media time	Positive smoking expectancies model	0.138 (0.029)	**(0.081, 0.196)**	**<.001**	0.009 (0.003)	**(0.003, 0.014)**	.002	**5.97% (1.27%, 10.67%)**	**.013**
	Negative smoking expectancies model	**0.153 (0.027)**	(0.100, 0.206)	**<.001**	0.001 (0.001)	(−0.002, 0.003)	.616	0.34% (−1.00%, 1.67%)	.617

*Note*: All models adjusted for age, sex, race/ethnicity, household income, parent education, depressive symptoms, Year 2 nicotine experimentation, Year 2 smoking expectancies (positive or negative, analyzed separately), and study site. The models assessing the direct effects of Year 2 social media time and Year 4 nicotine experimentation were also adjusted separately for Year 3 positive and negative smoking expectancies. Sampling weights were applied to yield representative estimates based on the American Community Survey from the US Census. Bold indicates statistical significance (*p* < .05).

Abbreviations: *B*, regression coefficient; CI, confidence interval; SE, standard error.

## DISCUSSION

In this large, demographically diverse sample of early adolescents from the ABCD Study, greater social media exposure was prospectively associated with a higher risk of nicotine experimentation two years later. This association was partially mediated by positive smoking expectancies, which explained 5.97% of the pathway. Although this percentage is modest, it is still concerning given the young age of participants and the link between early e‐cigarette exposure and later nicotine dependence,^3^ underscoring the potential life‐course impact of early influences that shape smoking expectancies.

These results align with prior research demonstrating social media exposure as a risk factor for adolescent nicotine experimentation.^31^ Longitudinal studies have found that frequent social media use and interactions with nicotine‐related content are linked to increased nicotine initiation and use.^3^, ^15^ Our study contributes to past literature by focusing specifically on early adolescents and identifying positive smoking expectancies as a significant mediating mechanism linking general social media exposure to subsequent nicotine experimentation.

The mediation results also align with a prior longitudinal study demonstrating that positive e‐cigarette expectancies mediated the association of exposure to e‐cigarette‐specific social media content and e‐cigarette use a year later among young adults.^31^ Although our measure captured overall social media exposure rather than nicotine‐specific content, both studies suggest that social media exposure may foster positive substance‐related beliefs that increase susceptibility to nicotine experimentation.^31^

Social Learning Theory provides a theoretical framework for the associations between social media use and nicotine experimentation, positing that behaviors are learned through observation, particularly in social contexts.^6^ Adolescents' exposure to depictions of nicotine use on social media may be associated with positive nicotine expectancies, including beliefs that it relieves stress, enhances social experiences, or reflects maturity.^5^ This is especially concerning with e‐cigarettes, as they tend to be marketed as safer and more socially acceptable.^32^ These beliefs may be associated with lower perceived risks and an increased willingness to try nicotine products.

Interestingly, we found no association or mediating effect of negative smoking expectancies. While health education and anti‐nicotine campaigns have shaped adolescents' beliefs of nicotine‐related harm,^33^ these influences may be less impactful in the context of social media. Negative expectancies may stem more from interventions outside of social media, such as in school or through public health messaging. Additionally, social media overwhelmingly depicts substance use, including nicotine use, in a positive light; in a review of 73 studies, 76.3% of substance‐related content was portrayed positively, while 20.2% was portrayed negatively.^9^ Therefore, social media exposure may be more commonly associated with positive rather than negative nicotine expectancies.

Limitations of this study include the use of self‐reported data, which may introduce reporting or social desirability bias. Moreover, participants who were excluded from the analytic sample due to missing data disproportionately represented racial and ethnic minority groups, which may introduce selection bias and limit generalizability. Additionally, the expectancy measures referenced cigarette smoking specifically, whereas the outcome captured experimentation with any nicotine product (including e‐cigarettes and smokeless tobacco). This construct mismatch may have attenuated the observed mediation effects since cigarette‐specific expectancies may not fully generalize to other nicotine products, such as e‐cigarettes, which are often perceived as less harmful. Additionally, the lack of data on specific social media content limited our ability to accurately assess participants' true exposure to nicotine‐specific content. While positive smoking expectancies partially mediated the relationship between social media exposure and nicotine experimentation, the relatively small mediating effect suggests that additional mechanisms may be involved. Factors such as advertisements, sensation seeking, problematic social media use, cyberbullying, and mental health could also mediate the relationship and warrant future investigation.^34^, ^35^ Strengths include a diverse national sample and a prospective study design focused on early adolescence, a critical window for intervention.

## CONCLUSION

This study suggests that greater social media exposure in early adolescence may be associated with positive beliefs about nicotine, potentially increasing the risk of nicotine experimentation. Social media regulatory efforts, such as improved age verification, content moderation, and platform‐specific policies, may help mitigate early initiation. Clinicians and public health professionals can support prevention by screening for nicotine‐related media exposure and addressing substance‐related beliefs during visits. Counter‐marketing campaigns on social media and efforts to promote media literacy may help challenge pro‐smoking beliefs and denormalize nicotine use among adolescents.

## CONFLICT OF INTEREST STATEMENT

The authors declare no conflicts of interest. The authors alone are responsible for the content and writing of this paper.

## Supporting information

Appendix A. Comparison of the sociodemographic characteristics of the Adolescent Brain Cognitive Development (ABCD) Study participants included vs. excluded in the analysis. Appendix B. 6‐Item Adolescent Smoking Consequences Questionnaire (ASCQ).
