# Supplementary material for: Social media use, smoking expectancies, and nicotine experimentation in early adolescents: A prospective cohort study
Source: Am J Addict. 2026 Feb 10;35(4):503–9. doi: 10.1111/ajad.70135 (PMC12990385; doi:10.1111/ajad.70135)
Supplement: Supplementary file 1 — Appendix A. Comparison of the sociodemographic characteristics of the Adolescent Brain Cognitive Development (ABCD) Study participants included vs. excluded in the analysis. Appendix B. 6‐Item Adolescent Smoking Consequences Questionnaire (ASCQ). [file AJAD-35-503-s001.docx]

| Appendix A. Comparison of the sociodemographic characteristics of the Adolescent Brain Cognitive Development (ABCD) Study participants included vs. excluded in the analysis | | | |
| --- | --- | --- | --- |
| Sociodemographic characteristics | Included  (n=8,292) | Excluded  (n=3,670) | p |
| Age (years) | 12.0 (0.7) | 12.1 (0.7) | **<0.001** |
| Sex |  |  | 0.606 |
| Female | 48.7% | 49.2% |  |
| Male | 51.3% | 50.8% |  |
| Race and ethnicity |  |  | **<0.001** |
| Asian | 5.3% | 6.0% |  |
| Black | 14.4% | 23.8% |  |
| Latino/Hispanic | 19.0% | 22.4% |  |
| Native American | 3.2% | 3.1% |  |
| Other | 1.3% | 1.8% |  |
| White | 56.7% | 42.9% |  |
| Household income |  |  | **<0.001** |
| $24,999 or less | 13.5% | 22.3% |  |
| $25,000 to $49,999 | 16.3% | 21.4% |  |
| $50,000 to $74,999 | 16.7% | 14.2% |  |
| $75,000 to $99,999 | 15.1% | 10.2% |  |
| $100,000 to $199,999 | 28.2% | 24.4% |  |
| $200,000 or greater | 10.3% | 7.5% |  |
| Parent's highest education |  |  | **<0.001** |
| High school education or less | 10.5% | 13.3% |  |
| College education or more | 89.5% | 86.7% |  |

| Appendix B. 6-Item Adolescent Smoking Consequences Questionnaire (ASCQ) | | | |
| --- | --- | --- | --- |
| Variable name | Items | Response options | Expectancy |
| ascq_section_q01 | Cigarettes help with concentration. | 1 = Never; 2 = Rarely; 3 = Sometimes; 4 = Often; 5 = Always | Positive |
| ascq_section_q02 | When someone is sad, smoking helps them feel better. | 1 = Never; 2 = Rarely; 3 = Sometimes; 4 = Often; 5 = Always | Positive |
| ascq_section_q03 | The look and feel of a cigarette in the mouth is good. | 1 = Never; 2 = Rarely; 3 = Sometimes; 4 = Often; 5 = Always | Positive |
| ascq_section_q04 | Parties are more enjoyable when a person is smoking. | 1 = Never; 2 = Rarely; 3 = Sometimes; 4 = Often; 5 = Always | Positive |
| ascq_section_q05 | Smoking will make a person cough. | 1 = Never; 2 = Rarely; 3 = Sometimes; 4 = Often; 5 = Always | Negative |
| ascq_section_q06 | Smoking makes people look ridiculous or silly. | 1 = Never; 2 = Rarely; 3 = Sometimes; 4 = Often; 5 = Always | Negative |
